# Influence of Smoking Consumption and Nicotine Dependence Degree in Cardiac Autonomic Modulation

**DOI:** 10.5935/abc.20160063

**Published:** 2016-06

**Authors:** Ana Paula Soares dos Santos, Dionei Ramos, Gabriela Martins de Oliveira, Ana Alice Soares dos Santos, Ana Paula Coelho Figueira Freire, Juliana Tiyaki It, Renato Peretti Prieto Fernandes, Luiz Carlos Marques Vanderlei, Ercy Mara Cipulo Ramos

**Affiliations:** 1 Departamento de Fisioterapia - Faculdade de Ciências e Tecnologia da Universidade Estadual Paulista (UNESP), Presidente Prudente, SP - Brazil; 2 Departamento de Clínica Médica - Faculdade de Medicina da Universidade de São Paulo (USP), Presidente Prudente, SP - Brazil; 3 Secretaria Municipal de Saúde de Presidente Prudente, Presidente Prudente, SP - Brazil

**Keywords:** Smoking, Tobacco Use / complications, Tobacco Use Disorders

## Abstract

**Background:**

Smoking consumption alters cardiac autonomic function.

**Objective:**

Assess the influence of the intensity of smoking and the nicotine dependence degree in cardiac autonomic modulation evaluated through index of heart rate variability (HRV).

**Methods:**

83 smokers, of both genders, between 50 and 70 years of age and with normal lung function were divided according to the intensity of smoking consumption (moderate and severe) and the nicotine dependency degree (mild, moderate and severe). The indexes of HRV were analyzed in rest condition, in linear methods in the time domain (TD), the frequency domain (FD) and through the Poincaré plot. For the comparison of smoking consumption, unpaired t test or Mann-Whitney was employed. For the analysis between the nicotine dependency degrees, we used the One-way ANOVA test, followed by Tukey's post test or Kruskal-Wallis followed by Dunn's test. The significance level was p < 0,05.

**Results:**

Differences were only found when compared to the different intensities of smoking consumption in the indexes in the FD. LFun (62.89 ± 15.24 vs 75.45 ± 10.28), which corresponds to low frequency spectrum component in normalized units; HFun (37.11 ± 15.24 vs 24.55 ± 10.28), which corresponds to high frequency spectrum component in normalized units and in the LF/HF ratio (2.21 ± 1.47 vs 4.07 ± 2.94). However, in the evaluation of nicotine dependency, significant differences were not observed (p > 0.05).

**Conclusion:**

Only the intensity of smoking consumption had an influence over the cardiac autonomic modulation of the assessed tobacco smokers. Tobacco smokers with severe intensity of smoking consumption presented a lower autonomic modulation than those with moderate intensity.

## Introduction

It is known that smoking is considered a serious public health problem with high incidence worldwide. It is estimated that there are 1.3 billion tobacco smokers in the world.^1^ Therefore, the consequences of the use of tobacco have, in the last few years,^2^ aroused the attention of researchers. The chronic use of tobacco creates tobacco-related diseases, the most common of which being related to the respiratory system.^3^ However, it is clear that smoking has an important extrapulmonary toxicity,^3^ which could represent serious risk factors for cardiovascular diseases and their respective complications, such as the damage of cardiac autonomic modulation.^4,5^

The changes that smoking causes in the cardiac autonomic modulation are thoroughly described in literature^6,7^ and can be evaluated through the heart rate variability (HRV),^8^ a non-invasive method, which describes the fluctuations between consecutive heartbeats.^9^ Eryonucu et al.^6^ found that smokers present lower rates of HRV, a result that is similar to those found by Barutcu et al.^7^ when assessing the HRV during controlled breathing exercises and muscle strength tests.

The intensity of smoking consumption, assessed by the number of cigarettes consumed per day, may influence the severity of the alterations observed in the autonomic modulation. Kupari et al.^10^ verified that individuals that smoked ten or more cigarettes per day presented greater impairment in cardiac autonomic modulation as compared to those who smoked less. Additionally, the risk of death for smokers increases according to the number of cigarettes smoked per day and the years of smoking.

The intensity of smoking consumption is strongly associated with the level of nicotine dependency, often times seen as the main determinant of the frequent use of cigarettes to avoid withdrawal symptoms.^11,12^ As a consequence of this more intense habit, the damages caused by smoking take bigger proportions.^12,13^

In spite of its importance, research in pertinent literature did not find studies that addressed the influence of nicotine dependency levels and smoking consumption in cardiac autonomic modulation. This represents a significant gap in the literature, considering that information of this nature could give smokers a more complete orientation on the importance of early cessation of this habit, as well as add elements of the exposed theme to the literature.

In this context, the objective of this study is to evaluate the influence of the intensity of smoking consumption and nicotine dependency degree on cardiac autonomic modulation through the index of HRV.

## Methods

### Population

Observational, cross-sectional study, in which 83 smokers were evaluated, determined by sample size calculation, with the LF/HF ratio as its variable. The magnitude of assumed significant difference was 1,8, considering a standard deviation of 1,19, based on a pilot study conducted with 80% beta-risk. The sample size, per evaluated group, resulted in 16 individuals of both genders, between 50 and 70 years of age, with normal lung function evidenced by spirometry. These individuals participated in a cessation program called PROCAT (Program of Anti-Tobacco Orientation and Awareness) of the University of Science and Technology Faculdade de Ciências e Tecnologias - FCT/UNESP, whose objective is the treatment of smokers through cognitive-behavioral and drug therapy.^13^

**Figure 1:**
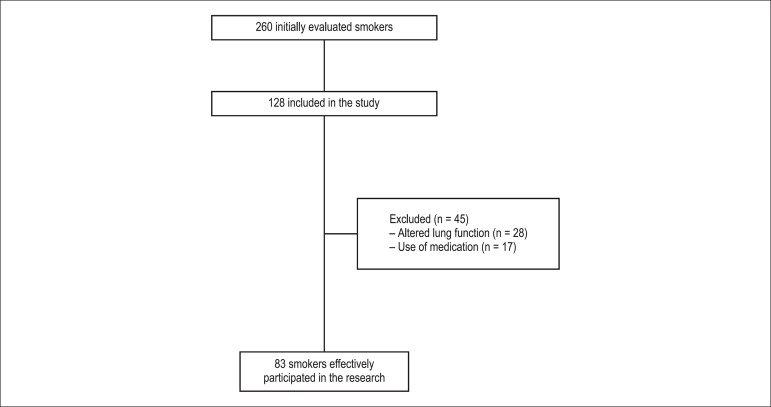
Flowchart of study losses.

This study did not include individuals who used narcotics or medications that influenced cardiac autonomic activity, alcoholics, or individuals with known diseases such as infections, metabolic or cardiorespiratory diseases. The flowchart of study losses is presented in Figure 1.

The volunteers were properly informed of the procedures and objective of this study. After agreement, they signed an informed consent to be part of the possible sample. This research was submitted to the appreciation of the Ethics Committee *FCT/UNESP* and by approved them (process n° 18/2011). All procedures were in accordance with Resolution 466/2012 of the National Health Counsil.

### Experimental Protocol

The protocol was carried out in the morning in order to soften the influences of the circadian rhythm, in a room with a controlled temperature of 23°C and relative air humidity between 50 and 60%. Before the evaluation, the individuals were asked to abstain from smoking, caffeine and physical activities for 12 hours prior to the execution of the protocol. The confirmation of the period of smoking abstinence was done through the uptake of carbon monoxide levels in exhaled air by using the Micro CO monoximeter (Micro Medical Limited, Rochester, England); values of under six parts per million (ppm) were considered to be abstinent.^14^

On the first day of the protocol, the characterization of the population was initially carried out through interviews with the volunteers to gather personal information, smoking habits (cigarettes per day and years of smoking to calculate packs/years)^15^ and degree of nicotine dependency. The characterization was concluded with the Fagerström questionnaire, which made it possible to separate the smokers into groups.

To separate the smokers according to their smoking consumption, the rate of packs/year was calculated by dividing the number of cigarettes smoked daily by 20 (number of cigarettes in a pack) and then multiplying that number by the years of smoking.^15^ Smokers were considered moderate when their smoking habits were between 10 and 20 packs per years and severe when that number surpassed 20 packs/year.^16^ Within the same degree of nicotine dependency, however, smokers were divided according to their scores in the Fagerström questionnaire, which consists of six questions that address some of the smoking habits such as the time of the first cigarette of the day, number of cigarettes throughout the day, discomfort for not being able to smoke in places where it is prohibited, satisfaction from smoking, frequency of smoking in the morning and illness occurrences. Each of these alternatives receives a score which allows the rating of three degrees of dependency: mild (0 to 3 points), moderate (4 to 6 points) and severe (7 to 10 points).^17^

Still on the first day of the protocol, anthropometric data was measured: weight (digital anthropometric scale W110 H - Welmy) and height (Stadiometer Standard Sanny) to calculate the Body Mass Index (BMI), and finally the lung function was calculated by using a portable spirometer (MIR - Spirobank - Italy) connected to a microcomputer. The criteria for the selection and analysis of the curves were in accordance with American Thoracic Society and European Respiratory Society.^18^ The values of normality were relative to the Brazilian population.^19^

On the second day of the protocol, the HRV was measured by capturing the heart rate (HR), beat by beat, using the cardiofrequencimeter Polar S810i. A chest strap for the capturing of HR was placed at the level of the xiphoid process of the sternum and an HR receptor strap was placed on the wrist to record the received data. After being fit with the equipment, the volunteers were asked to stay seated for 20 minutes, resting, breathing spontaneously.^20,21^

### Analysis of the indexes of heart rate variability

To analyse the indexes of HRV, 256 RR intervals selected from the most stable part of the chart were used after digital filtering, completed by manual filtering to eliminate artifacts and ectopic beats; only series with over 95% of sinus beats were included in the study. The analysis was processed by the software Kubios (University of Kuopio, Finland).^22^

In the time domain (TD), the duration of RR intervals and the indexes RMSSD (Root Mean Square of Successive Differences) and SDNN (Standard Deviation of Normal to Normal intervals) were used, both expressed in milliseconds (ms). In the frequency domain (FD), there was use of the low frequency spectrum component (LF, 0.04 - 0.15 Hz), which represents sympathetic and parasympathetic activity, with predominance of high frequency and sympathetic (HF, 0.15 - 0.40 Hz), this represents parasympathetic activity, in absolute values (ms^2^) and in normalized units (un), as well as the LF/HF ratio.^23,24^ The spectral analysis was calculated using the fast Fourier transform algorithm.^8^

The Poincaré plot was also used for the analysis of the HRV. The plot represents, graphically, a correlation between consecutive RR intervals, in which each point is represented - on the horizontal axis X (abscissa) by the previous normal RR interval, and on the vertical axis Y (ordinate) by the following RR interval - and it may be analysed quantitatively and qualitatively through the assembly of an ellipse formed by the graphical representation of the RR intervals. The center of this ellipse is determined by the average of the RR intervals.^25,26^

For the quantitative analysis of the plot, through the adjustment of the ellipse of the shape formed by the attractor, the following indexes were calculated: SD1 (standard deviation of the instantaneous beat to beat variability); SD2 (standard deviation of the long-term continuous R-R intervals); and the SD1/SD2 ratio, which shows the ratio between short and long-term variations of the RR intervals.^9,27^

The qualitative plot analysis was done through the analysis of the shapes formed by its attractor. The following patterns were considered: I) a shape in which an increase in the dispersion of RR intervals is observed with an increase in intervals was considered characteristic of a normal plot; II) a shape with little beat-to-beat global dispersion and without an increase in the dispersion of long-term RR intervals was considered characteristic of a plot with smaller variability.^28^

### Statistical Analysis

To analyse the data, the statistical program Graphpad Prism® was used. The normal distribution of data was assessed through the Shapiro-Wilk test, and the description of the results was done as mean values ± standard deviation or median [interquartile intervals 25-75%]. To analyse the different intensities of smoking consumption, the unpaired t test or Mann-Whitney test was used, depending on the normality of the data. For the different degrees of nicotine dependency, the One-way ANOVA followed by Tukey's test or Kruskal-Wallis' test followed by Dunn's test were used, also depending on the normality of the data. Significance level used in the study: p < 0.05.

## Results

### Characteristics of individuals and lung funtion

Table 1 presents the personal, anthropometric and spirometric data of the smokers, separated according to intensity of smoking consumption. The groups were similar in relation to BMI and lung function. Statistically significant differences were found between moderate and severe smokers when the groups were compared by age, cigarettes smoked per day, years of smoking and packs/year.

**Table 1:** Characterization of smokers divided according to intensity of smoking consumption in relation to age, BMI, spirometric values and smoking habits, expressed in mean ± standard deviation and median [Interquartile interval 25 – 75%]

Variables	Moderate smokers	Severe smokers	p
N	34	49	
**Anthropometry**			
Gender (M/F)	(7/27)	(29/20)	
Age (years)	53.76 ± 4.14*	56.10 ± 4.74	0.0213
	52.00 [50.00 – 56.25]	56.00 [52.00 – 59.00]	
BMI (kg/m^2^)	26.46 ± 4.84	26.12 ± 4.72	0.7673
	26.54 [22.28 – 29.94]	26.00 [22.24 – 28.84]	
**Spirometric values**			
FEV_1_ (% Pred)	95.40 ± 11.33	95.68 ± 8.30	0.9112
	96.52 [86.00 – 104.30]	95.07 [90.58 – 99.89]	
FVC (% Pred)	99.26 ± 12.47	97.81 ± 8.18	0.5851
	102.50 [87.14 – 108.80]	97.08 [91.14 – 103.20]	
FEV_1_/FVC	78.38 ± 4.60	78.46 ± 6.21	0.9560
	78.50 [75.35 – 82.15]	77.65 [73.85 – 83.28]	
FEF_25-75%_ (% Pred)	91.74 ± 22.93	96.97 ± 29.23	0.4571
	92,65 [78.20 – 105.00]	89.78 [77.81 – 116.30]	
**Smoking consumption history**			
Time of smoking (years)	28.79 ± 7.85*	38.31 ± 7.46	< 0.0001
	29.00 [20.00 – 35.50]	38.00 [33.00 – 42.50]	
Cigarettes/day	12.82 ± 4.59*	22.55 ± 6.77	< 0.0001
	10.00 [10.00 – 16.25]	20.00 [20.00 – 20.00]	
Packs/year	17.05 ± 3.30*	42.74 ± 13.34	< 0.0001
	18.63 [14.75 – 20.00]	40.00 [30.75 – 50.00]	

N: number of volunteers; M: male; F: female; BMI: body mass index; kg: kilogram; - m: meter; FEV_1_: forced expiratory volume in the first second; FVC: forced vital capacity; FEV_1_/FVC: ratio between FEV_1_ and FVC; FEF_25-75%_: forced expiratory flow between 25 and 75% of FVC;

(*) Statistically significant difference in comparison to severe smokers.

Table 2 presents the personal, anthropometric and spirometric data of the smokers, separated according to nicotine dependency. The groups were similar as related to age, BMI and lung function. In the Fagerström questionnaire, according to the score obtained, there was statistically significant difference between the groups only in relation to nicotine dependency.

**Table 2:** Characterization of smokers devided according to nicotine dependency in relation to age, BMI, spirometric values and score in the Fagerström questionnaire, expressed in mean ± standard deviation and median [Interquartile interval 25 – 75%]

**Variables**	**Mild smokers**	**Moderate smokers**	**Severe smokers**	**p**
N	18	33	32	
**Anthropometry**				
Gender (M / F)	(5 / 13)	(15 / 18)	(16 / 16)	
Age (years)	56.06 ± 5.63	55.36 ± 3.75	54.41 ± 4.87	0.4043
	56.50 [50.00 – 60.25]	55.00 [52.00 – 59.00]	53.00 [50.25 – 57.00]	
BMI (kg/m^(2^)	25.19 ± 5.03	26.90 ± 4.94	26.22 ± 4.38	0.4977
	25.36 [21.94 – 26.99]	26.46 [22.31 – 30.68]	26.62 [22.46 – 28.63]	
**Spirometric values**				
FEV_1_ (% Pred)	94.05 ± 13.30	93.60 ± 8.06	98.57 ± 8.19	0.1629
	93.21 [83.47 – 104.30]	94.10 [88.43 – 98.65]	98.12 [92.42 – 105.80]	
FVC (% Pred)	98.30 ± 11.98	97.11 ± 10.67	99.86 ± 8.41	0.6472
	98.18 [89.99 – 107.90]	97.42 [87.31 – 104.30]	100.20 [91.34 – 105.80]	
FEV_1_/FVC%	77.08 ± 5.27	77.82 ± 6.12	79.85 ± 4.99	0.2816
	76.60 [73.75 – 80.60]	77.20 [73.45 – 82.75]	79.60 [77.00 – 83.60]	
FEF_25-75%_ (% Pred)	87.05 ± 28.98	89.20 ± 22.64	105.30 ± 27.33	0.0586
	90.15 [60.04 – 105.00]	87.58 [77.26 – 100.90]	95.08 [85.32 – 123.90]	
**Smoking Dependency**				
Fagerström (SCORE)	2.66 ± 0.84^(†^	5.48 ± 0.61*	7.84 ± 1.01	< 0.0001
	3.00 [3.00 – 3.00]	6.00 [5.00 – 6.00]	7.50 [7.00 – 8.75]	

N: number of volunteers; M: male; F: female; BMI: body mass index; kg: kilogram; m: meter; FEV_1_: forced expiratory volume in the first second; FVC: forced vital capacity; FEV_1_/FVC: ratio between FEV_1_ and FVC; FEF_25-75%_: forced expiratory flow between 25 and 75% of FVC;

(*) Statistically significant difference in comparison to severe smokers.

(†) Statistically significant difference in comparison to moderate and severe smokers.

### Indexes of HRV of smokers according to the intensity of smoking consumption and degree of nicotine dependency

Table 3 depicts the indexes of HRV of the smokers, divided according to the intensity of smoking consumption. Statistically significant differences were found in the LF and HF indexes un, LF/HF ratio, and SD1/SD2 ratio

**Table 3:** HRV indexes evaluated in the different groups of smokers according to the intensity of smoking consumption expressed in mean ± standard deviation and median [Interquartile interval 25 – 75%]

Variables	Moderate smokers	Severe smokers	p
N	34	49	
RR (ms)	819.40 ± 173.00	831.40 ± 145.50	0.6467
	828.00 [743.50 – 885.80]	828.00 [743.50 – 885.80]	
SDNN (ms)	30.47 ± 12.77	31.20 ± 13.79	0.5943
	29.00 [22.75 – 34.25]	31.00 [20.00 – 41.50]	
RMSSD (ms)	23.61 ± 9.54	21.01 ± 11.03	0.1538
	22.45 [16.23 – 29.53]	18.70 [13.40 – 26.90]	
LFms^(2^	77.82 ± 115.20	104.20 ± 138.40	0.3617
	43.00 [25.00 – 86.25]	58.00 [23.50 – 128.00]	
HFms^(2^	39.68 ± 49.93	32.69 ± 43.95	0.0776
	23.50 [14.25 – 48.00]	16.00 [7.00 – 39.00]	
LFun	62.89 ± 15.24*	75.45 ± 10.28	< 0.0001
	64.85 [54.33 – 74.23]	77.30 [66.30 – 82.50]	
HFun	37.11 ± 15.24*	24.55 ± 10.28	< 0.0001
	35.15 [25.78 – 45.68]	22.70 [17.50 – 33.70]	
LF/HF	2.21 ± 1.47*	4.07 ± 2.94	0.0002
	1.84 [1.19 – 2.89]	3.40 [1.96 – 4.72]	
SD1 (ms)	16.99 ± 6.85	15.12 ± 7.90	0.1473
	16.25 [11.65 – 21.08]	13.40 [9.55 – 19.30]	
SD2 (ms)	47.03 ± 20.01	48.30 ± 21.41	0.7354
	46.70 [32.85 – 53.43]	46.70 [31.10 – 58.50]	
SD1/SD2	0.38 ± 0.13*	0.31 ± 0.11	0.0204
	0.35 [0.27 – 0.45]	0.29 [0.23 – 0.36]	

N : number of volunteers; ms: milliseconds; SDNN: Standard Deviation of Normal to Normal intervals; RMSSD: Root Mean Square of Successive Differences; LF: low frequency; un: normalized unit; HF: high frequency; SD1: standard deviation of the instantaneous beat to beat variability; SD2: standard deviation of the long-term continuous R-R intervals;

(*) Statistically significant difference in comparison to severe smokers.

Table 4 depicts the HRV indexes of the smokers divided into groups, according to the degree of nicotine dependency. No significant differences were found in the analysed indexes.

**Table 4:** Indexes of HRV evaluated in the different groups of smokers according to the degree of nicotine dependency expressed in mean ± standard deviation and median [Interquartile interval 25 – 75%]

Variables	Mild smokers	Moderate smokers	Severe smokers	p
N	18	33	32	
RR (ms)	844.70 ± 82.51	840.5 ± 171.40	811.80 ± 179.10	0.6632
	839.00 [788.00 – 937.00]	868.00 [782.00 – 888.00]	824.50[732.30 – 892.30]	
SDNN (ms)	29.78 ± 11.10	32.18 ± 14.16	30.69 ± 13.57	0.9287
	30.00 [24.75 – 36.00]	31.00 [22.50 – 40.00]	29.00 [20.50 – 40.25]	
RMSSD (ms)	21.78 ± 7.44	23.68 ± 10.16	20.82 ± 12.10	0.3369
	22.25 [17.23 – 26.98]	22.40 [15.40 – 30.45]	18.50 [13.53 – 26.15]	
LFms^(2^	75.22 ± 56.72	108.20 ± 172.90	88.72 ± 105.20	0.9648
	56.50 [21.75 – 131.00]	50.00 [24.00 – 101.50]	45.00 [24.25 – 118.80]	
HFms^2^	28.11 ± 21.93	41.12 ± 43.32	34.31 ± 58.25	0.2748
	22.00 [14.25 – 36.50]	24.00 [8.50 – 56.00]	15.50 [8.25 – 36.50]	
LFun	66.72 ± 11.07	68.81 ± 14.61	73.72 ± 14.35	0.0630
	68.35 [60.13 – 75.80]	71.10 [60.45 – 80.05]	78.30 [65.53 – 83.00]	
HFun	33.28 ± 11.07	31.19 ± 14.61	26.28 ± 14.35	0.0630
	31.65 [24.20 – 39.88]	28.90 [19.95 – 39.55]	21.70 [17.00 – 34.48]	
LF/HF	2.34 ± 1.19	3.02 ± 2.15	4.14 ± 3.32	0.0628
	2.16 [1.50 – 3.13]	2.46 [1.53 – 4.04]	3.61 [1.90 – 4.88]	
SD1 (ms)	15.71 ± 5.40	17.04 ± 7.27	14.98 ± 8.65	0.3330
	16.15 [12.33 – 19.65]	16.10 [11.05 – 21.80]	13.40 [9.65 – 18.83]	
SD2 (ms)	47.24 ± 19.07	49.88 ± 20.89	46.64 ± 21.47	0.7365
	46.50 [37.48 – 53.28]	48.60 [32.10 – 60.45]	45.80 [30.60 – 54.48]	
SD1/SD2	0.34 ± 0.08	0.35 ± 0.13	0.32 ± 0.13	0.3203
	0.34 [0.28 – 0.39]	0.33 [0.25 – 0.42]	0.29 [0.23 – 0.38]	

N: number of volunteers; ms: milliseconds; SDNN: Standard Deviation of Normal to Normal intervals; RMSSD: Root Mean Square of Successive Differences; LF: low frequency; un: normalized unit; HF: high frequency; SD1: standard deviation of the instantaneous beat to beat variability; SD2: standard deviation of the long-term continuous R-R intervals.

### Qualitative analyses of the Poincaré plot

**Figure 2:**
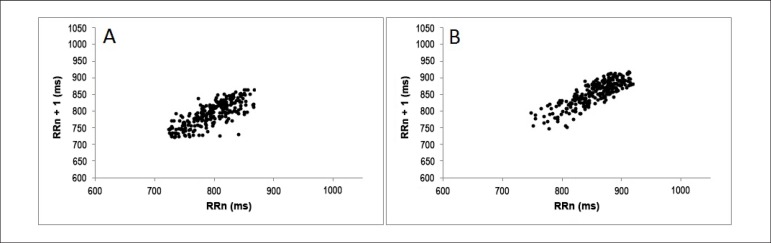
Qualitative analysis of the Poincaré plot in the different intensities of smoking consumption: moderate (individual A - SD1: 16,9 and SD2: 47) and severe (individual B - SD1: 15,2 and SD2: 50,4).

**Figure 3:**
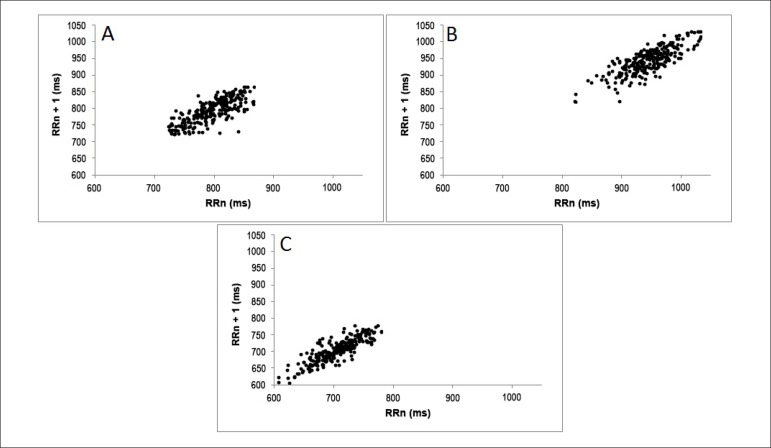
Qualitative analysis of the Poincaré plot in the different degrees of nicotine dependency: mild (individual A - SD1: 16.9 and SD2: 47), moderate (individual B - SD1: 17.8 and SD2: 52.9) and severe (individual C - SD1: 13.4 and SD2: 46.7).

The qualitative analyses of the Poincaré plot is expressed in figures 2 and 3, which show standard examples of the plot in smokers that presented SD1 and SD2 index values close to the mean, according to the intensity of smoking consumption and the degree of nicotine dependency, respectively.

## Discussion

The present study evaluated the influence of smoking consumption and degree of nicotine dependency over cardiac autonomic modulation of smokers by using HRV indexes. The main results showed that smoking consumption alone had influence over the cardiac autonomic modulation of the assessed smokers. In the indexes that describe the HRV in the FD, the LFun index and the LF/HF ratio were increased in severe smokers, as opposed to the HFun index, which was significantly smaller in this group. This characterizes a sympathetic predominance in severe smokers, in comparison to moderate smokers. Carcigi et al.^29^ found an enlarged LF/HF ratio in smokers with a consumption of over 20 cigarettes/day in comparison to non-smokers. Baructu et al.^7^ observed that the length of smoking consumption showed a positive correlation with the LF/HF ratio, which characterizes a smaller vagal modulation and larger sympathetic modulation the longer the length of smoking consumption.

In the quantitative of the Poincaré plot, the SD1/SD2 ratio, which represents the ratio between the long and short-term variations of records of RR intervals, was significantly larger in moderate smokers, who, when compared to severe smokers, had better HRV.

The qualitative Plot analysis did not show differences in the dispersion of RR intervals. However, the analyses of the plot of the different degrees of nicotine dependency showed that mild and moderate smokers present larger RR intervals when compared to severe smokers, but without significant differences. Reduced RR intervals, like the ones found in severe smokers, suggest a higher HR in resting in these individuals, which may be more predisposed to the surging of cardiovascular events.^30^ The HR may have a direct effect on the cardiovascular system, because it increases myocardial consumption of oxygen and induces fatigue, in addition to being associated with higher pressure levels.^30^

In this study, the studied population is considered between adults and seniors, between 50 and 70 years old, which may justify, in part, the obtained results in the analysed HRV indexes. The results show that the participants in the severe smokers group, separated by the intensity of smoking consumption, were older than the ones in the moderate smokers group. Literature shows that there is an influence of age in the autonomic modulation, that is, the older the individual, the higher the sympathetic action that can be observed; so this factor may have influenced the observed results.^31,32^

Age is an important determinant in autonomic modulation, with aging being associated to a progressive cardiac vagal decline as age advances,^31^ which may be considered a limitation in the present study. Paschoal et al.^32^ found a reduction of the indicative values of parasympathetic activity and an increase in cardiac sympathetic activity, as from the 5^th^ decade of life, in healthy individuals, when compared to younger individuals. Hering et al.^33^ showed that the autonomic responses depend on age in smokers as well and may result from alterations in the responses of the adrenal medulla, reduced clearance of norepinephrine and/or inhibition of the process of norepinephrine reabsorption, caused by chronic exposure to smoking.

The biggest chronicity of smoking was shown to be related to lower vagal activity and higher sympathetic activity, as verified in other studies,^7,10,29^ which characterize the decrease of HRV indexes in smokers.^6^ The reduction of HRV may be associated to health damages, and is a concerning factor associated to the increase in mortality and morbidity in several conditions.^9^

No differences were found in the cardiac autonomic modulation of the evaluated smokers, when comparing different degrees of nicotine dependency. This non-difference may support the evidence that personality traits may be more strongly associated to the dependency than the smoking itself.^34^ Some authors are investigating the association between nicotine dependency and psychiatric disturbances such as depression, anxiety, schizophrenia, among others.^35,36^ Such evidence may appear from the assumption that, in the Fagerström questionnaire, only one question addresses the quantity of cigarettes smoked, per day, by the individual, while the others are related to his/her behavior.

As a limitation of the study, the lack of a control group consisting of non-smoking individuals, and of tests to detect asymptomatic heart diseases may be pointed out. These factors could have contributed to a better understanding of the obtained results.

## Conclusion

Only the intensity of smoking consumption had influences over cardiac autonomic modulation of the evaluated smokers. Smokers with severe smoking consumption intensity presented worse autonomic modulation than moderate ones.

## References

[1] Saleheen D, Zhao W, Rasheed A (2014). Epidemiology and public health policy of tobacco use and
cardiovascular disorders in low- and middle-income countries. Arterioscler Thromb Vasc Biol.

[2] Prado GF, Lombardi EM, Morais AM, Martins SR, Santos U de P (2012). Smoking what has been addressed in Brazilian
journals. Arq Bras Cardiol.

[3] Yanbaeva DG, Dentener MA, Creutzberg EC, Wesseling G, Wounters EF (2007). Systemic effects of smoking. Chest.

[4] Manzano BM, Vanderlei LC, Ramos EM, Ramos D (2010). Smoking implications on cardiac autonomic control. Arq Ciênc Saúde.

[5] Middlekauff HR, Park J, Moheimani RS (2014). Adverse effects of cigarette and noncigarette smoke exposure on
the autonomic nervous system mechanisms and implications for cardiovascular
risk. J Am Coll Cardiol.

[6] Eryonucu B, Bilge M, Guler N, Uzun K, Gencer M (2000). Effects of cigarette smoking on the circadian rhythm of heart
rate variability. Acta Cardiol.

[7] Barutcu I, Esen AM, Kaya D, Turkmen M, Karakaya O, Melek M (2005). Cigarette smoking and heart rate variability dynamic influence of
parasympathetic and sympathetic maneuvers. Ann Noninvasive Electrocardiol.

[8] Rajendra Acharya U, Paul Joseph K, Kannathal N, Lim CM, Suri JS (2006). Heart rate variability a review. Med Bio Eng Comput.

[9] Vanderlei LC, Pastre CM, Hoishi RA, Carvalho TD, Godoy MF (2009). Basic notions of heart rate variability and its clinical
applicability. Rev Bras Cir Cardiovasc.

[10] Kupari M, Virolainen J, Koskinen P, Tikkanen MJ (1993). Short-term heart rate variability and factors modifying the risk
os coronary artery disease in a population sample. Am J Cardiol.

[11] Shiffman S, Ferguson SG, Dunbar MS, Scholl SM (2012). Tobacco dependence among intermittent smokers. Nicotine Tob Res.

[12] Park S, Lee JY, Song TM, Cho SI (2012). Age-associated changes in nicotine dependence. Public Health.

[13] Freire AP, Ramos D, Silva BS, David RM, Pestana PR, Fernandes RA (2014). Results of smoking cessation program analysis of new
procedures. ConScientiae Saúde.

[14] Santos UP, Gannam S, Abe JM, Esteves PB, Filho MF, Wakassa TB (2001). Emprego da determinação de monóxido de
carbono no ar exalado para a detecção do consumo de
tabaco. J Pneumol.

[15] Sociedade Brasileira de Pneumologia e Tisiologia (2002). Diretrizes para testes de função
pulmonar. J Pneumol.

[16] Nagelmann A, Tonnov Ä, Laks T, Sepper R, Prikk K (2011). Lung dysfunction of chronic smokers with no signs of
COPD. COPD.

[17] Fagerström K, Russ C, Yu C-R, Yunis C, Foulds J (2012). The Fagerström Test for Nicotine Dependence as a predictor
of smoking abstinence a pooled analysis of verenicline clinical trial
data. Nicotine Tob Res.

[18] Miller MR, Hankinson J, Brusasco V, Burgos F, Casaburi R, Coates A, ATS/ERS Task Force (2005). Standardization of spirometry. Eur Respir J.

[19] Neder JA, Andreoni S, Castelo-filho A, Nery LE (1999). Reference values for lung function tests I. Static
volumes. Braz J Med Biol Res.

[20] Gamelin FX, Berthoin S, Bosquet L (2006). Validity of the polar S810 heart rate monitor to measure R-R
intervals at rest. Med Sci Sports Exerc.

[21] Vanderlei LC, Silva RA, Pastre CM, Azevedo FM, Godoy MF (2008). Comparison of the polar S810i monitor and the ECG for the
analysis of heart rate variability in the time and frequency
domains. Braz J Med Biol Res.

[22] Tarvainen MP, Niskanen JP, Lipponen JA, Ranta-Aho PO, Karjalainen PA (2014). Kubios HR-heart rate variability analysis
software. Comput Methods Programs Biomed.

[23] Ribeiro JP, Moraes RS (2005). Heart rate variability as a tool for the investigation of the
autonomic nervous system. Rev Bras Hipertens.

[24] Rassi A (2001). Compreendendo melhor as medidas de análise de
variabilidade da freqüência cardíaca. J Diag Cardiol.

[25] Kitlas A, Oczeretko E, Kowalewski M, Borowska M, Urban M (2005). Non linear dynamics methods in the analysis of the heart rate
variability. Annales Academicae Medicae Bialostocensis.

[26] Niskanen JP, Tarvainen MP, Ranta-aho PO, Karjalainen PA (2004). Software for advanced HRV analysis. Comput Methods Programs Biomed.

[27] Manzano BM, Vanderlei LC, Ramos EM, Ramos D (2011). Acute effects of smoking on autonomic modulation analysis by
Poincaré plot. Arq Bras Cardiol.

[28] Tulppo MP, Mäkikallio TH, Seppänen T, Laukkanen RT, Huikuri HV (1998). Vagal modulation of heart rate during exercise effects of age and
physical fitness. Am J Physiol.

[29] Cagirci G, Cay S, Karakurt O, Eryasar N, Kaya V, Canga A (2009). Influence of heavy cigarette smoking on heart rate variability
and heart rate turbulence parameters. Ann Noninvasive Electrocardiol.

[30] Perret-Guillaume C, Joly L, Benetos A (2009). Heart rate as a risk factor for cardiovascular
disease. Prog Cardiovasc Dis.

[31] Zhang J (2007). Effect of age and sex on heart rate variability in healthy
subjects. J Manipulative Physiol Ther.

[32] Paschoal MA, Volanti VM, Pires CS, Fernandes FC (2006). Variabilidade da frequência cardíaca em diferentes
faixas etárias. Rev bras fisioter.

[33] Hering D, Somers VK, Kara T, Kucharska W, Jurak P, Bieniaszewski L (2006). Sympathetic neural responses to smoking are age
dependent. J Hypertens.

[34] Rondina RC, Botelho C, Silva AMC, Gorayeb R (2003). Psychological profile and nicotine dependence in smoking
undergraduate students of UFMT. J Pneumol.

[35] Breslau N, Kilbey MM (1991). Nicotine dependence, major depression, and anxiety in young
adults. Arch Gen Psychiatry.

[36] Herran A, de Santiago A, Sandoya M, Fernandez MJ, Diez-Manrique JF (2000). Determinants of smoking behavior in outpatients with
schizophrenia. Schizophr Res.

